# Prognostic implications of the interaction between intratumoral microbiome and immune response in gastric cancer

**DOI:** 10.1128/spectrum.02830-24

**Published:** 2025-04-09

**Authors:** Sifan Liu, Lingyu Qi, Lu Dong, Wenjing Sun, Siying Liu, Peng Li, Nan Zhang

**Affiliations:** 1 Department of Gastroenterology, State Key Laboratory for Digestive Health, National Clinical Research Center for Digestive Diseases, Beijing Digestive Disease Center, Beijing Key Laboratory for Precancerous Lesions of Digestive Diseases, Beijing Friendship Hospital, Capital Medical University 26455, Beijing, China; 2 School of Clinical Medicine, Shandong Second Medical University 372527 , Weifang, China; University of Arkansas for Medical Sciences, Little Rock, Arkansas, USA

**Keywords:** gastric cancer, intratumoral microbiome, host–microbiome interaction, gastrointestinal microbiome, tumor immune microenvironment

## Abstract

Gastric cancer (GC) prognosis is significantly influenced by intratumoral microbiomes, which modulate host–immune interactions. This study analyzed data from the The Cancer Genome Atlas (TCGA) and Gene Expression Omnibus (GEO) databases to identify immune genes associated with GC prognosis and conducted prognostic immune subtypes. GC patients were classified into two distinct prognostic immune phenotypes C1 and C2 based on the non-negative matrix factorization consensus clusters. Phenotype C2 exhibited a better prognosis and distinct immune characteristics, including enhanced presence of Th2 and Th17 cells and improved response to chemotherapy. In contrast, phenotype C1 showed higher expression levels of PDCD1LG2 and TLR9, which were critical immune factors involved in immune regulation. Both phenotypes were linked to immune genes influencing intratumoral microbiomes and GC immunotherapy responses. A prediction risk model was constructed using the LASSO regression analysis and showed great prognostic value for GC patients. The key genes were correlated with immune cells and suppressed the function of the host immune system. The intratumoral microbiomes were strongly associated with the hosts’ immune infiltration and significantly interacted with host immune genes to influence GC outcomes. *Candidatus Nitrosotenuis* plays a significant role in predicting the prognosis of GC patients. This research underscores the pivotal role of intratumoral microbiomes in GC prognosis and supports the development of future personalized therapeutic approaches.

**IMPORTANCE:**

Increasing evidence confirms the presence of intratumoral microbiomes. However, the role of the intratumoral microbiomes in the progression of gastric cancer and their relationship with the immune microenvironment remain unclear. Our study classified gastric cancer patients into two immune prognostic subtypes, C1 and C2, using non-negative matrix factorization consensus clusters. The C2 subtype exhibited a better prognosis and more pronounced immune characteristics. Microbiome analyses revealed associations between both subtypes and immune genes that affect intratumoral microbiomes and their responses to immunotherapy. The intratumoral microbiomes were closely linked with host immune infiltration and significantly interacted with immune genes, which influence the prognosis of gastric cancer. Notably, *Candidatus Nitrosotenuis* showed a significant prognostic value in gastric cancer patients. Our findings highlight the critical role of the intratumoral microbiomes in affecting gastric cancer prognosis and its interaction with the immune microenvironment, supporting future personalized therapeutic approaches.

## INTRODUCTION

Gastric cancer (GC) ranks as the fifth most common malignancy worldwide and the fourth leading cause of cancer-related deaths largely due to its poor prognosis stemming from late-stage diagnosis (1, 2). High incidence rates are seen in East Asia, Eastern Europe, and parts of Central and South America (3). Factors including family history, age, diet, *Helicobacter pylori* (*H. pylori*) (4), and Epstein–Barr virus contribute to GC development (5). Early GC is often asymptomatic, whereas advanced stages may present nausea and abdominal pain. Early detection and intervention are imperative for improving the prognosis and the survival quality of individuals afflicted with GC (6).

Research shows that the microbiome in the tumor microenvironment (TME) influences tumorigenesis (7–9). *H. pylori* and other intratumoral microbiomes contribute to GC pathogenesis and affect treatment outcomes (10). An emerging area of investigation in GC research is the intratumoral microbiomes, with a particular focus on the interactions between bacteria and immunity-related genes. Studies have identified a distinct microbial profile in GC compared to normal tissues, which may influence carcinogenesis and tumor progression through immune modulation and inflammation (11–13). However, the role of bacterium-immunity genes in GC is still unclear, requiring further research to understand their impact on cancer development and treatment. This deeper understanding could lead to new strategies for GC prevention, diagnosis, and treatment.

This study profiles immune genes in GC to explore prognostic differences, immune phenotypes, and responses to immunotherapy. Understanding the interplay between intratumoral microbiota and GC molecular features could improve immunotherapeutic strategies and microbial modulation, enhancing GC treatment efficacy and patients’ qualities of life.

## MATERIALS AND METHODS

### Data acquisition

We utilized The Cancer Genome Atlas (TCGA) database, the largest cancer gene information repository, to access mRNA expression data from stomach adenocarcinoma (STAD) patients, including 36 normal and 412 tumor samples. We also downloaded Series Matrix Files from National Center for Biotechnology Information (NCBI) Gene Expression Omnibus (GEO) for GSE84437 (433 STAD patients, GPL6947), GSE66229 (300 STAD patients, GPL570), and GSE26901 (109 STAD patients, GPL6947) containing complete expression profiles and survival data. Additionally, we retrieved 955 relevant genes (relevance score > 1) from the GeneCards database for our analysis. We retrieved 664 stomach neoplasm-related microRNAs (miRNAs) from the Human MicroRNA Disease Database (http://www.cuilab.cn/hmdd).

### Classification of immune subtypes

Immune-related genes were used as the total candidate gene set. Cox regression analysis was subsequently performed using the R package “survival” to evaluate the correlation of all candidate genes with overall survival (OS). Subsequently, the unsupervised non-negative matrix factorization (NMF) clustering method was performed using the NMF package, and the method was applied to three external validation sets using the same candidate genes from the GEO database (GSE26901, GSE66229, and GSE84437). The *k* value at which the correlation coefficient begins to decrease was selected as the optimal number of clusters, and the mRNA expression data of the above immune genes were used to verify the subtype assignment.

### Immune cell infiltration

The Microenvironment Cell Populations (MCP) algorithm was used to assess 10 cell populations, including eight immune cell populations (T cells, CD8^+^ T cells, natural killer cells, cytotoxic lymphocytes, B-cell lineage, monocyte lineage, myeloid-like dendritic cells, and neutrophils) and two stromal cell populations (endothelial cells, fibroblasts). Additionally, single-sample gene set enrichment analysis (ssGSEA) was employed to determine the gene set enrichment scores in individual samples. GSVA-R software estimated six more immune cell types (Tregs, Th1, Th2, Th17, central memory T cells, and Tem). The ESTIMATE algorithm was also applied to compute immune and stromal scores, reflecting the abundance of these cell types of gene characteristics.

### Subtype GSEA

Using the limma package, the log_2_ [fold change (FC)] values for each gene across subtypes were obtained. The cluster Profiler R package was then employed to perform the GSEA analysis on Gene Ontology (GO) and Kyoto Encyclopedia of Genes and Genomes (KEGG) pathways. Finally, the top 10 subtype-specific upregulated pathways based on the highest normalized enrichment score (NES) values were selected for presentation. The gene set was obtained from the MSigDB database (http://www.gsea-msigdb.org/gsea/downloads.jsp).

### Performance verification of immune subtypes

Differentially expressed genes (DEGs) between GC subtypes were analyzed using the limma package, with the selection criteria for differential genes set at an adjusted *P*-value < 0.05. Genes exhibiting significant differential expression in all possible comparisons were considered subtype-specific. From these, the top 30 genes with the highest log_2_FC values in each subtype were selected to construct a predictive model, resulting in a 60-gene classifier. The Nearest Template Prediction (NTP) algorithm was then applied to predict subtypes based on the 60-gene data set, and the results were compared with classifications derived from the NMF algorithm.

### Immunotherapy and drug sensitivity analysis

We predicted immunotherapy efficacy by comparing subclass gene expression profiles to immunotherapy-treated samples using SubMap analysis. We also used the pRRophetic R package on the Genomics of Drug Sensitivity in Cancer (GDSC) database to estimate chemotherapy sensitivity, calculating IC50 for each drug, validating our predictions through 10-fold cross-validation, adjusting for batch effects, and averaging duplicate gene expressions.

### Differences in the intratumoral microbiome between immune subtypes

The data were collected using Kraken analysis to study the intratumoral microbiome (14). The Kraken algorithm is a fast and highly accurate program for assigning taxonomic labels to metagenomic sequences using k-mer alignments. The immune subtypes were further predicted and verified through interaction analysis of the immune infiltration microbiome, gene expression microbiome, and immunotherapy microbiome. The genes mainly included immune genes, cytotoxic T lymphocyte (CTL) genes (15), and key genes.

### Regulatory network analysis of important genes

The R package “RcisTarget” was employed to predict transcription factors, calculating NES based on motif data. We enhanced motif annotations using similarity and gene sequence information. We determined motif overexpression by computing the area under the curve (AUC) for each motif–gene set pair and derived NES from the AUC distribution of all motifs. The rcistarget. hg19.motifdb. cisbpont.500bp database was used for gene–motif rankings.

### Multiplex immunohistochemistry

Multiplex immunohistochemistry (mIHC) was performed using the following protocol: after processing the tissue sections with xylene and a gradient of ethanol, antigen retrieval was conducted using microwave heating. Following blocking, the sections were incubated with primary antibodies. Then, the sections were incubated with horseradish peroxidase (HRP)-conjugated secondary antibodies at room temperature for 20 min, subsequently followed by the addition of a tyramide signal amplification (TSA) reagent for signal amplification. Each of these steps (blocking, primary antibody incubation, HRP-conjugated secondary antibody incubation, and TSA signal amplification) was repeated for each marker. Finally, 4′,6-diamidino-2-phenylindole staining was performed, and the sections were mounted. Imaging was conducted using the 3DHISTECH Imaging System (Budapest, Hungary). The primary antibodies used included: CXCR4 (1:300, 60042-1-Ig, Proteintech), GPR35 (1:100, 55248-1-AP, Proteintech), CD4 (1:1,000, ab288724, Abcam), and CD8 (1:1,000, ab245118, Abcam). Data analysis was performed using HALO software (v3.3, New Mexico, USA). The use of these pathological tissue sections was approved by the Medical Ethical Committee of Beijing Friendship Hospital Affiliated to the Capital Medical University (2018-P2-058).

### Cell culture and transfection

The mouse GC cell line MFC (iCell-m035) was obtained from iCell Bioscience, Inc. (Shanghai, China) and authenticated by STR analysis within the last 6 months. MFC cells were cultured in RPMI 1640 (Gibco, Waltham, MA, USA) containing 10% fetal bovine serum (Gibco) in a 37°C, 5% CO_2_ incubator. Mycoplasma contamination was assessed every 2 months using a mycoplasma detection kit (C0297S, Beyotime Biotechnology). The plasmids for mouse CXCR4, GPR35, and LMNB2 were purchased from Youbio (Changsha, China) and transfected according to the instructions provided with Lipofectamine 2000 (11668019, Thermo Fisher Scientific) or Megatran 2.0 (TT210003, Origene).

### Model construction and prognosis

Four key genes were selected, and LASSO regression was used to build a prognosis model, calculating the patient risk scores based on gene expression and regression coefficients. Patients were categorized into low- and high-risk groups by the median risk score, with survival differences assessed via Kaplan–Meier analysis and log-rank test. We evaluated the risk score’s prognostic role through stratified analysis and assessed model accuracy with receiver operating characteristic (ROC) curves.

### Fluorescence *in situ* hybridization

The independent GC prognosis tissue microarray used in this study was purchased from Biotechwell (Shanghai, China). Detailed prognostic information is provided in Table S1. A Texas Red-labeled probe targeting *Candidatus Nitrosotenuis* strains was synthesized by TinyGene (Shanghai, China), while a CY5-labeled EUB338 probe was used as a positive control to identify bacterial colonization in tissue specimens. The fluorescence *in situ* hybridization (FISH) assay was performed according to the manufacturer’s instructions for the direct fluorescence bacterial *in situ* hybridization detection kit (Toyobo, Japan), and fluorescence staining of the tissue sections was subsequently observed under a confocal microscope.

### Animal study

All animals used in this study were provided by Capital Medical University (Beijing, China) and maintained under specific pathogen-free conditions. Six week-old male NOD/SCID and C57 BL/6 mice were purchased from Beijing Vital River Laboratory Animal Technology Co., Ltd. and used for tumorigenesis experiments. All mice were randomly divided into four groups (*n* = 3). An equal number of transfected MFC cells was subcutaneously injected into NOD/SCID and C57 BL/6 mice. The mice were sacrificed 10 days after inoculation, and tumor volumes were calculated using the formula length × width² / 2, while tumor weights were also recorded.

### Statistical analysis

Survival curves were generated by the Kaplan‒Meier method and compared by log-rank tests. In this analysis, R language (version 4.0) was used. All the statistical tests were two-sided, and *P* < 0.05 was considered to indicate statistical significance.

## RESULTS

### Identification of prognosis-related genes and molecular subtypes

**Fig 1:**
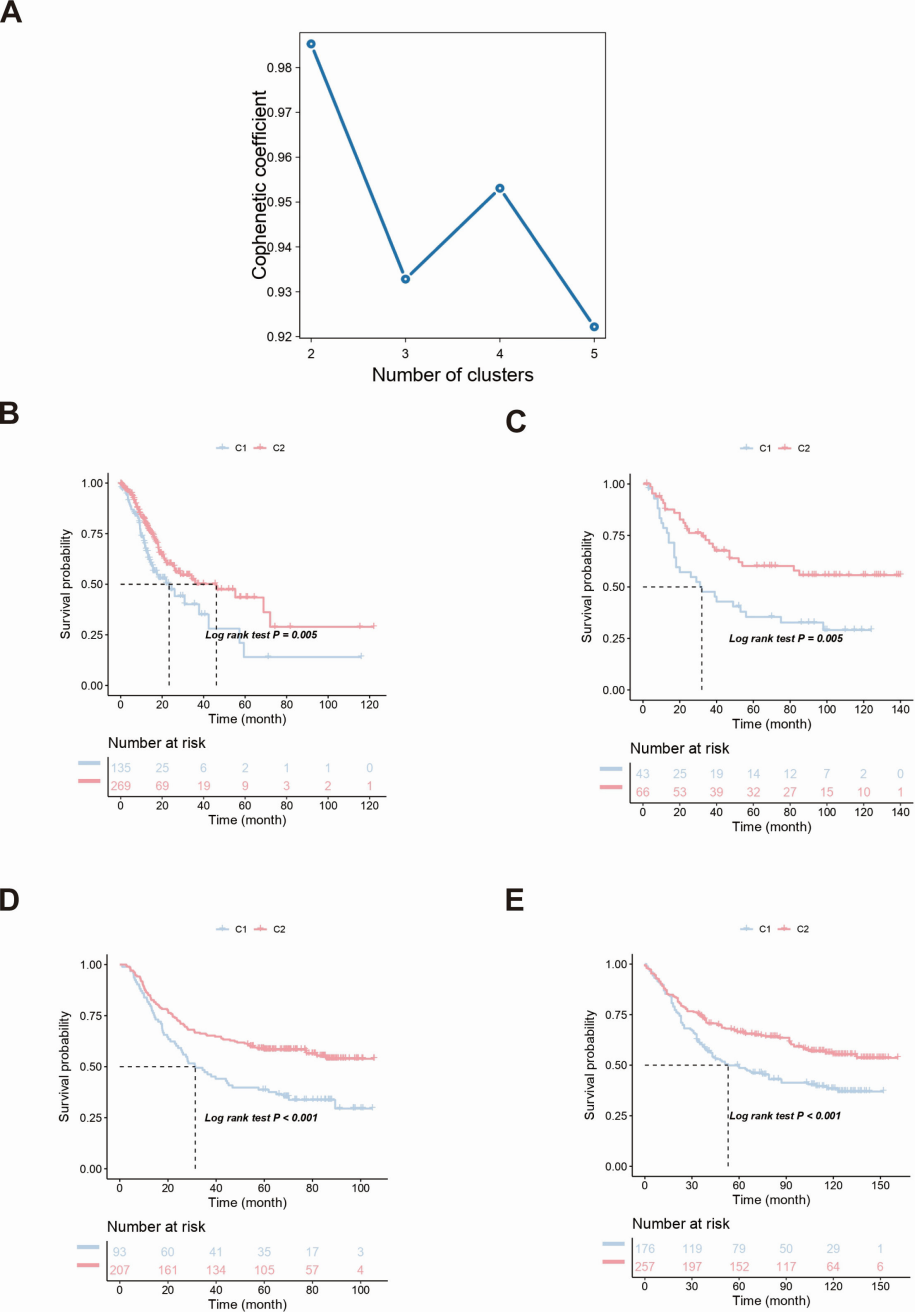
Subtype clustering and survival analysis. (**A**) Optimal clustering of subtypes was performed based on the immune genes. (**B**) Survival analysis was performed in the TCGA database. (**C–E**) Survival analyses were performed in the GSE26901 (**C**), GSE66229 (**D**), and GSE84437 (**E**) data sets.

To determine the prognostic genes in the immune gene set, we collected the clinical information of STAD patients and used the Cox single-factor regression feature selection algorithm to screen out the characteristic genes in GC. A total of 57 prognosis-related genes were identified through Cox single-factor regression (*P* < 0.05) analysis. We used the NMF consensus clustering method to cluster the TCGA data set containing GC samples based on the expression profiles of the above 57 candidate genes and determined the optimal *k* value. After comprehensive consideration, we selected *k* = 2 as the optimal cluster number (Fig. 1A). Subsequent independent validation on a data set of GC samples from three external validation set databases using the previously mentioned *k* = 2 classifications similarly revealed two distinct molecular subtypes. We observed significant prognostic differences in the TCGA data set, with C2 having a better survival possibility than C1 (Fig. 1B). In addition, similar differences were also observed between the subtypes of the three validation sets, with the OS of C2 being significantly greater than that of the C1 subgroup (Fig. 1C through E).

### Analysis of the immune characteristics of subtypes

**Fig 2:**
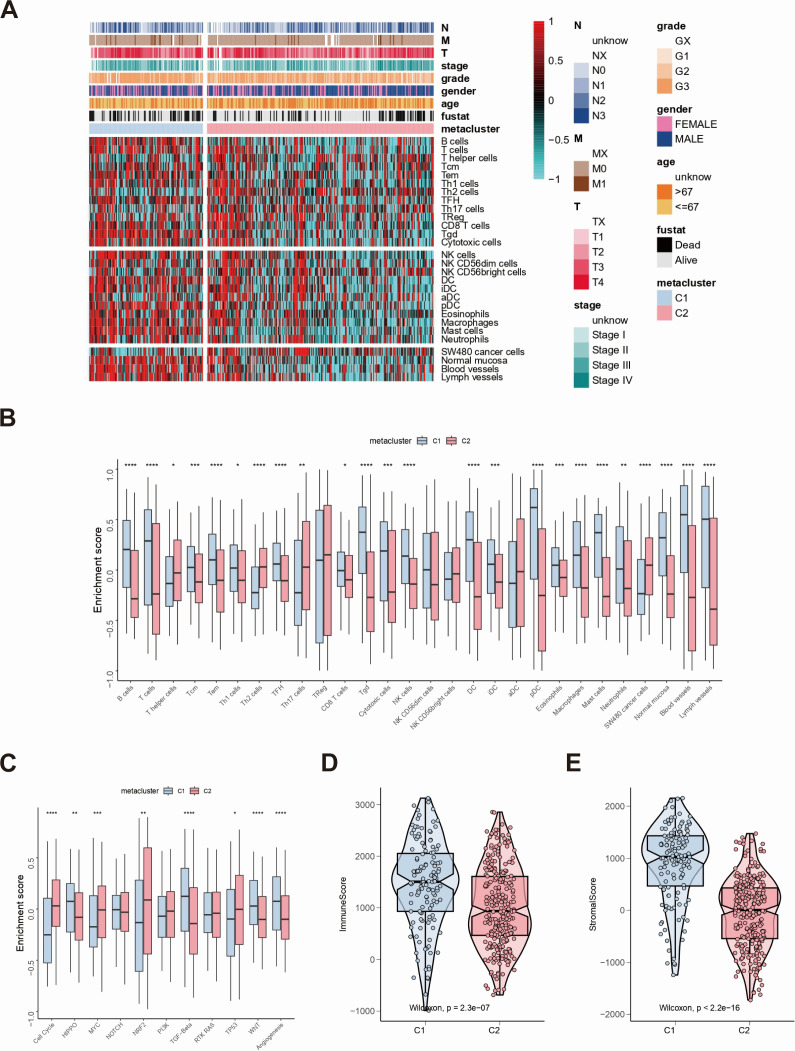
Analysis of immune characteristics of subtypes. (**A**) Heatmap of the correlation between subtype clinical characteristics and 28 immune features was made. (**B**) Differential expression of subtypes and 28 immune-related cells was validated. (**C**) Differential expression of subtypes and pathways was performed. (**D and E**) Immune (**D**) and stromal (**E**) score characteristics between subtypes were compared.

Considering that the classification was based on immune-related genes, whether different subtypes have different immune characteristics was further explored. First, the immune process was quantified using the ssGSEA algorithm (Fig. 2A), and then differential analysis was performed to discover subtype-specific immune signatures. The results revealed differences in immune characteristics between the two subgroups, and the immune characteristics of the C2 subgroup, such as T helper and Th2 cells, were significantly greater than those of the C1 subgroup (Fig. 2B). In addition, C2 had higher cell cycle and MYC pathway activation-related scores than C1 (Fig. 2C). The study then used the ESTIMATE algorithm to calculate immune and stromal scores. There were differences in immune and stromal scores between the two groups, and the immune and stromal scores of C2 were significantly lower than those of C1 (Fig. 2D and E).

### Evaluation of immune levels by the MCP and ssGSEA algorithms

**Fig 3:**
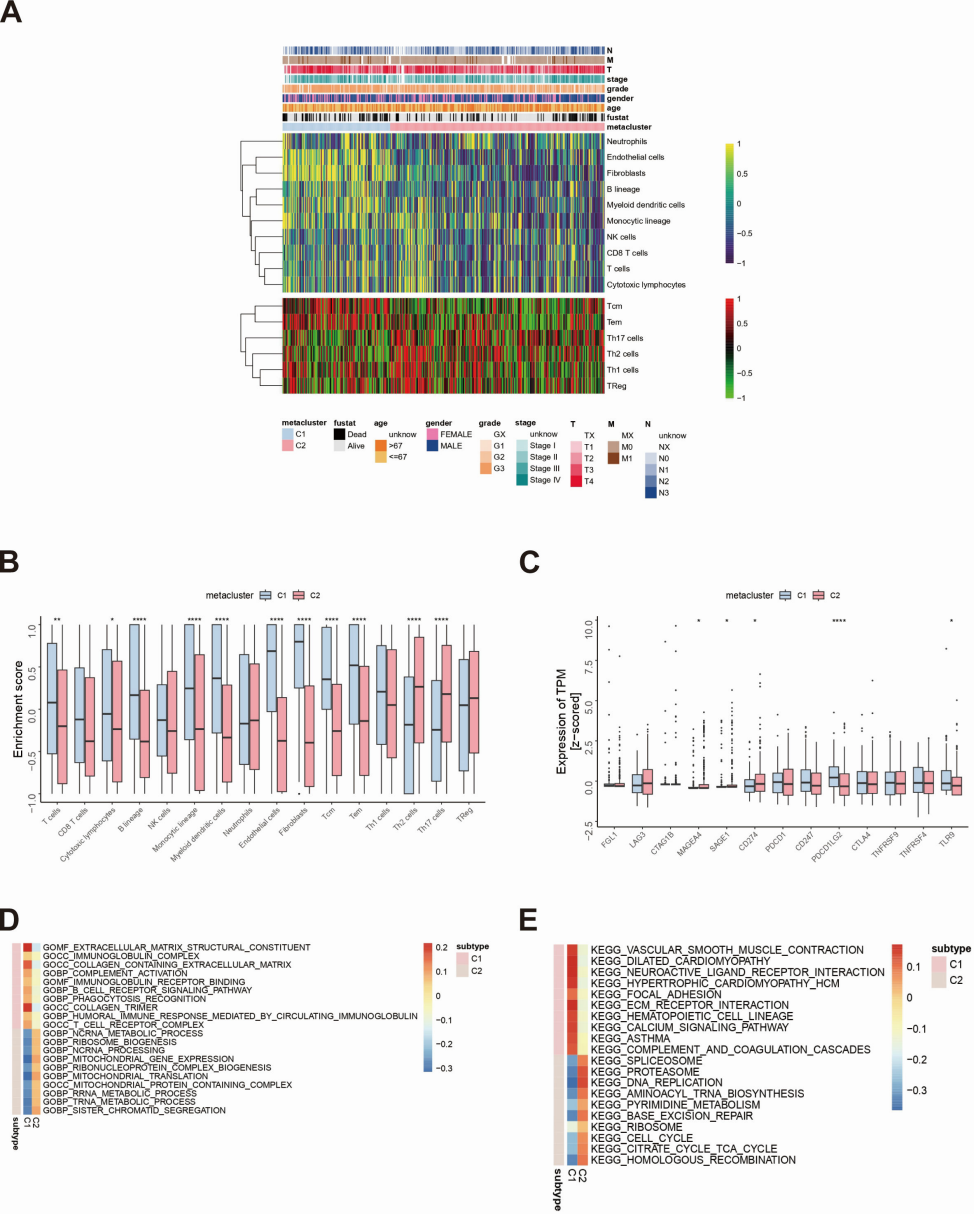
Immune characteristics of MCP and enrichment analysis of subtypes. (**A**) Heatmap of subtype clinical features and 16 types of immune-related cells was performed. (**B**) Differential expressions of subtypes and 16 types of immune-related cells were conducted. (**C**) Differential expressions of subtypes and immune factors were carried out. GO (**D**) and KEGG (**E**) analyses of the two subtypes were performed.

Since there are significant differences in immune scores between subtypes, the MCP and ssGSEA algorithms were further used to conduct in-depth research on immune gene-concentrated immune infiltration. The abundances of 16 immune-related cell types were calculated using the MCP counter and the ssGSEA algorithm and presented in a heatmap (Fig. 3A). The results showed significant differences in immune infiltration levels between the two subtypes, suggesting the reproducibility of the classified immune signatures. In addition, there were significantly more Th2 and Th17 cells in the C2 subpopulation than in the C1 subpopulation, and the results for the Tcm and Tem subpopulations were consistent with previous immune-related feature enrichment results (Fig. 3B). Further investigation of the associations between subtypes and the expression of 12 potentially targetable immune factors selected based on current drug inhibitors in clinical trials or already approved for specific cancer types revealed that the expression of the PDCD1LG2 and TLR9 increased in C1 (Fig. 3C).

We further used the ssGSEA algorithm to quantitatively analyze the GO and KEGG pathways and found that there were multiple related pathways with obvious differences between the two subgroups. In the C2 subtype, the pathways with higher scores in the GO analysis were rRNA metabolic process, tRNA metabolic process, sister chromatid segregation, and other pathways (Fig. 3D). According to the KEGG analysis, the pathways with higher C2 subtype scores were mainly related to pathways, such as cell cycle, citrate cycle tricarboxylic acid (TCA) cycle, and homologous recombination (Fig. 3E). According to the GO analysis, the pathways with higher C1 subtype scores were mainly the extracellular matrix structural constituent, immunoglobulin complex, and other pathways (Fig. 3D). According to the KEGG analysis, the pathways with higher C1 subtype scores were mainly the vascular smooth muscle contraction and dilated cardiomyopathy pathways (Fig. 3E). The pathways enriched in the two subtypes and their distinct functionalities may mediate the differences in clinical prognosis observed between the two subtypes.

### Drug susceptibility and mutation load level verification of molecular subtypes

**Fig 4:**
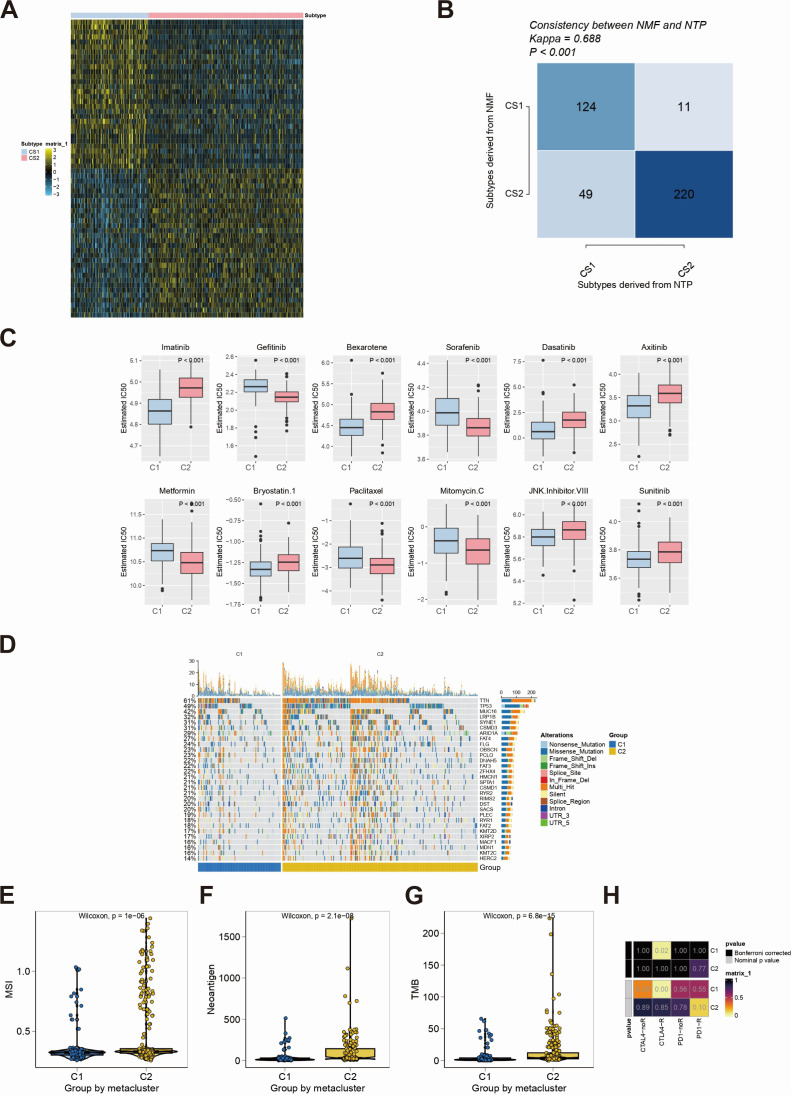
Drug sensitivity relatedness and tumor mutation of subtypes. (**A**) The 60-gene classifier was generated and visualized. (**B**) The performance of non-negative matrix factorization (NMF) subtypes was evaluated by the Nearest Template Prediction (NTP) algorithm. (**C**) Subtype analysis of sensitivity to chemotherapeutic agents was made. (**D**) The top 30 genes with higher mutation frequencies in single nucleotide polymorphism-related data of gastric cancer were visualized. (**E through G**) Comparison of differences in microsatellite instability (MSI) (**E**), neoantigen (**F**), and tumor mutational burden (TMB) (**G**) between subgroups was made. The prediction of immunotherapy between subtypes using SubMap was carried out (H).

After a comprehensive consideration of accuracy and clinical application potential, the top 30 genes with the largest log_2_FC values in each subtype were selected for the development of subtype classification. Therefore, a 60-gene classifier was generated and visualized (Fig. 4A). The performance of the NMF subtypes was evaluated by the NTP algorithm (Fig. 4B). Based on the drug sensitivity data of the GDSC database, we used the R package “pRRophetic” to predict the chemotherapy sensitivity of each tumor sample and further explored the sensitivity of subtypes and common chemotherapy drugs. The results showed that subtypes were significantly related to patient sensitivity to drugs, such as imatinib, sorafenib, bexarotene, gefitinib, dasatinib, and axitinib (Fig. 4C). We further explored the mutational profiles of patients in different subtypes, and the results showed that the proportion of patients with mutations in multiple genes, such as TTN in group C2, was significantly greater than that in group C1 (Fig. 4D). On the contrary, in multiple group comparisons, the results showed that microsatellite instability (MSI), neoantigen, and tumor mutational burden (TMB) were significantly different between subtypes (Fig. 4E through G). In addition, based on the immunotherapy data set, the sensitivity of the two subgroups to antitumor immunotherapy was predicted. The results showed that the C1 subtype was more sensitive to immunotherapy (Fig. 4H).

### Immune-related gene validation of molecular subtypes

**Fig 5:**
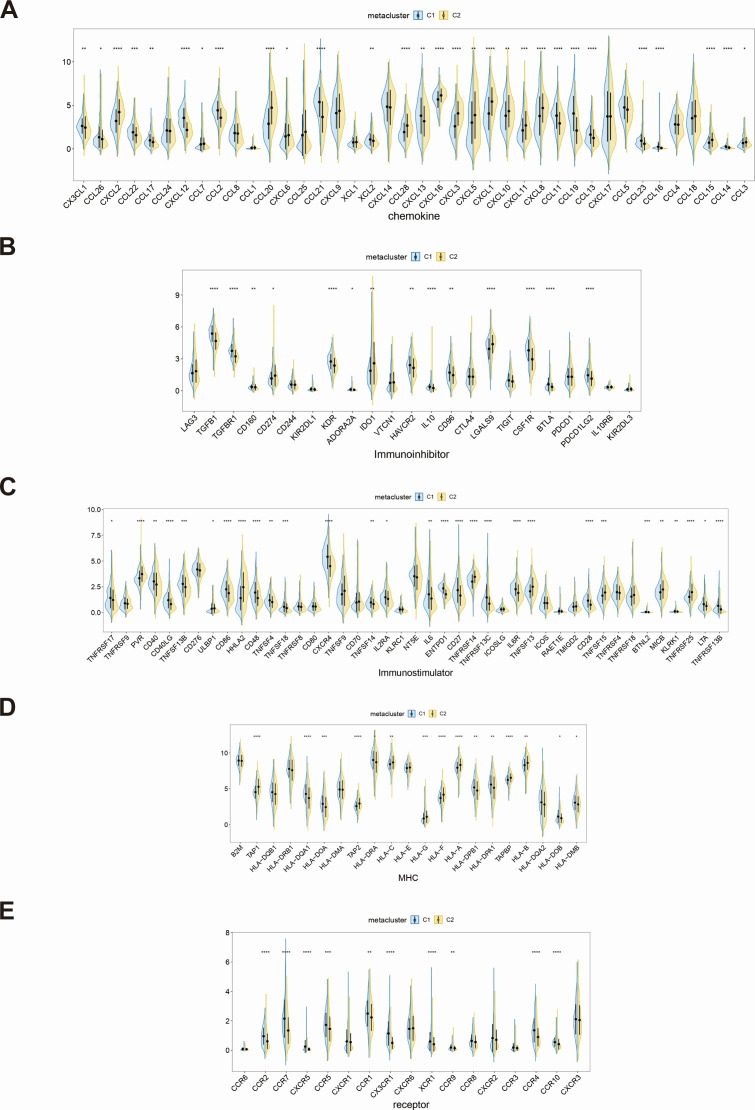
Differences in immune-related gene sets between subtypes. Differences of chemokines (**A**), immunoinhibitors (**B**), immunostimulators (**C**), major histocompatibility complex (MHC) (**D**), and receptors (**E**) between subtype groups were determined.

We conducted a comprehensive extraction of multiple immune-related gene sets from the TISIDB database, which included information on immune regulators, chemokines, and cell surface receptors. Subsequent analysis revealed that the expression profiles of a substantial cohort of immune-related genes exhibited significant differences across various immune subtypes, as delineated in Fig. 5A through E. In the C1 subtype, the expression levels of immune-related genes, such as CXCL12, KDR, CD27, HLA-DQA1, and CCR7, are significantly higher, while in the C2 subtype, genes, such as CXCL2, LGALS9, HHLA2, and TAP1, exhibit elevated expression levels. These findings highlight the differential expression of immune-related genes between the two subtypes.

### TIDE analysis between molecular subtypes

**Fig 6:**
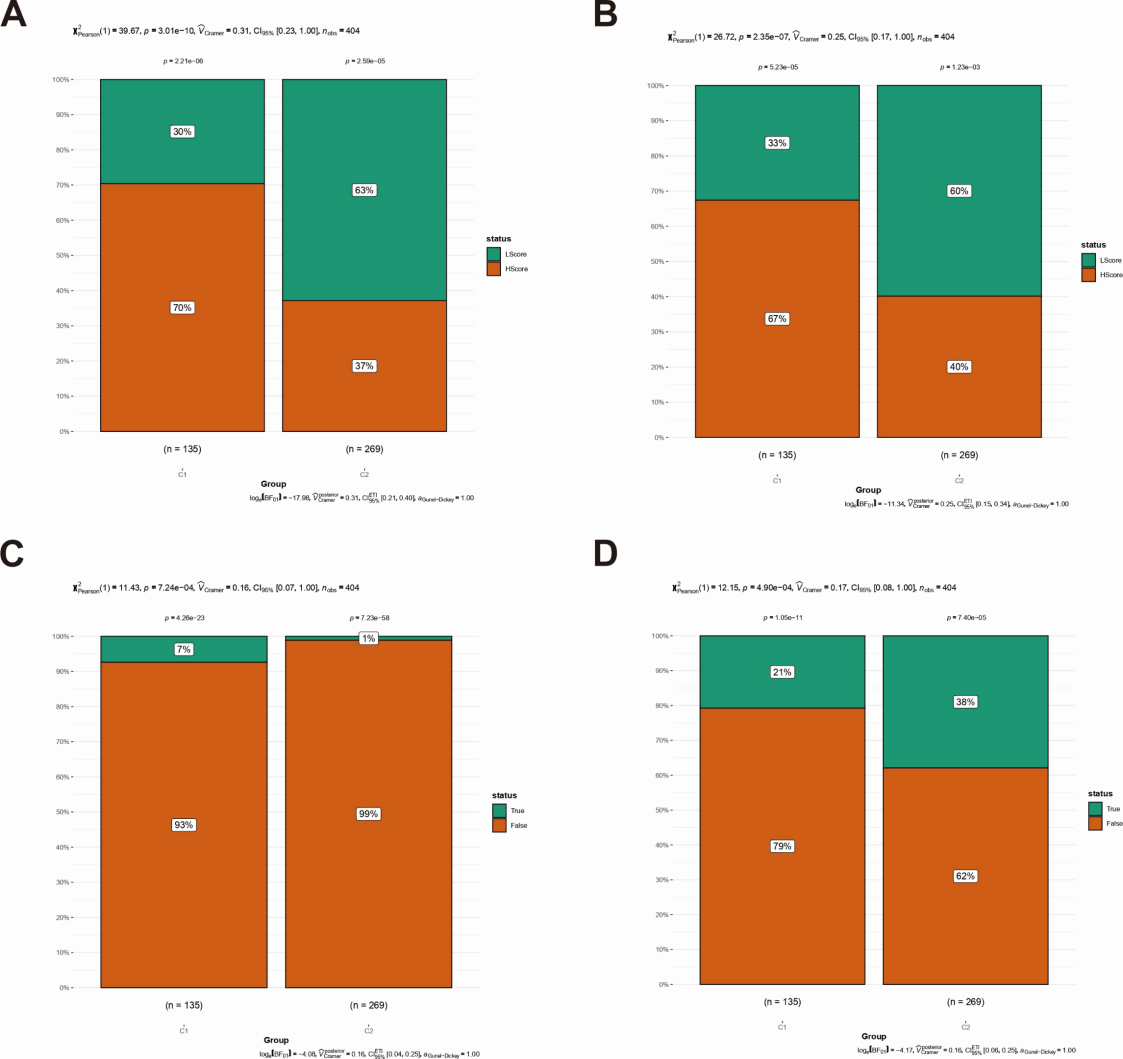
Prediction of immune therapy response in subtypes. Differences of dysfunction (**A**), exclusion (**B**), no benefits (**C**), and responder (**D**) between subtype groups based on the TIDE database predictions were explored.

Tumor immune dysfunction and exclusion (TIDE) analysis serves as a methodological tool to evaluate the intricate interactions between the immune system and tumor cells within the TME, as well as the processes by which the immune system recognizes and responds to neoplastic cells. The objective of this analysis was to elucidate why the immune system fails to mount an effective attack and eradicate tumor cells in certain contexts and to investigate strategies to activate or potentiate the immune response against tumors through immunotherapeutic interventions. The TIDE analysis revealed differences between subtypes, among which dysfunction, exclusion, no benefits, and responder were significantly different between subtypes (Fig. 6A through D).

### Intratumoral microbiome distribution between subtypes

**Fig 7:**
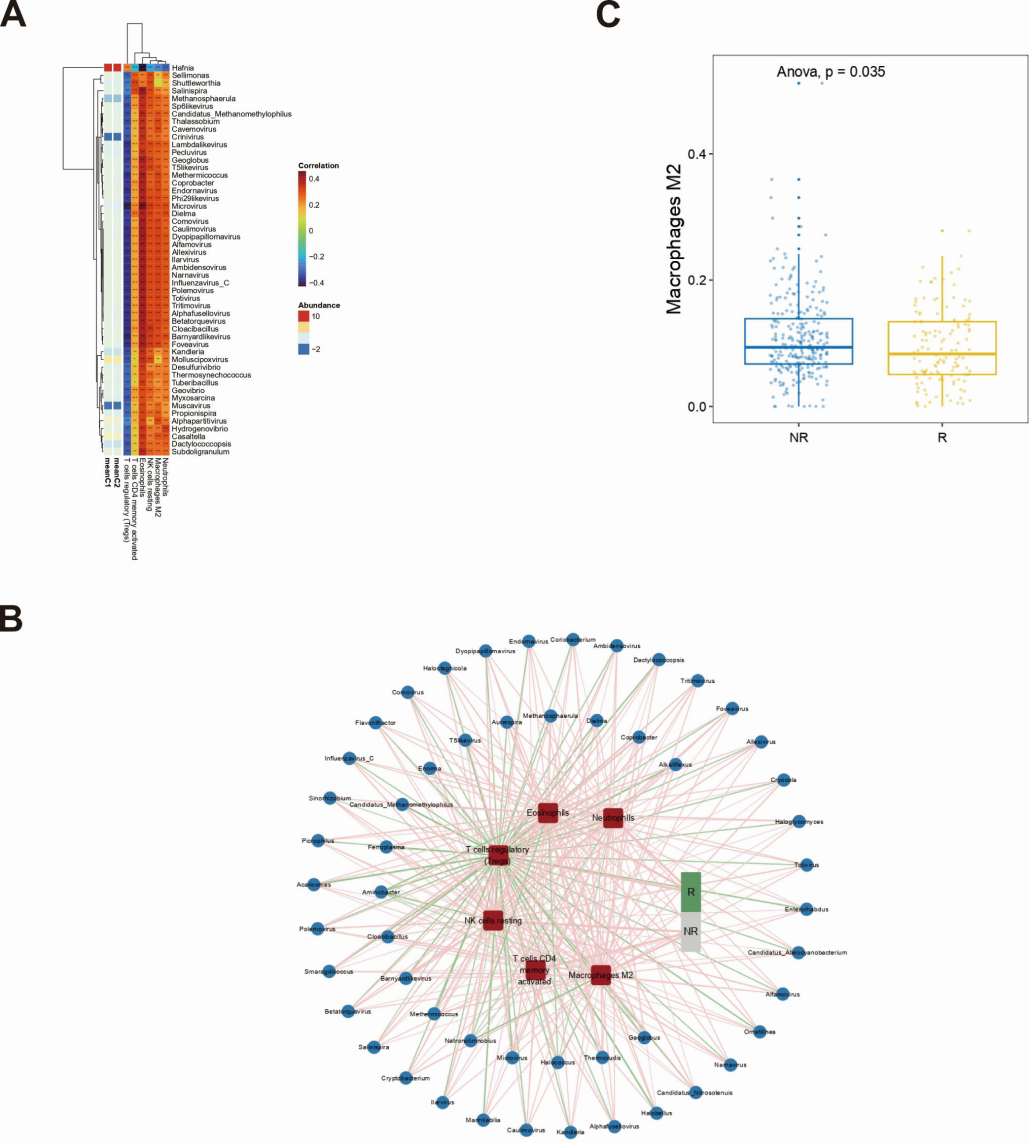
Microbial distributions and immune infiltration between subtypes. (**A**) The correlation between immune infiltration and intratumoral bacteria was performed. (**B**) The impact of immunotherapy on immune infiltration and intratumoral microbiome was validated. (**C**) Differential expressions of macrophage M2 were explored between the response and nonresponse groups.

The influence of the intratumoral microbiomes on the immune microenvironment warrants further investigation. Pearson correlation analysis was performed between microbial abundance and immune infiltration level. The results showed that the abundances of eosinophils and natural killer cells resting were significantly positively correlated with microbiomes, such as *Betatorquevirus*, *Cloacibacillus*, and *Barnyard-like virus*, while the abundances of regulatory T cells (Tregs) were significantly negatively correlated with the abundances of microbiomes, such as *Ambidensovirus*, *Narnavirus*, *Influenza virus_C*, and *Polemovirus* (Fig. 7A). At the same time, correlation analysis between immune-related genes and microorganisms was conducted for the C1 and C2 subtypes. This indicates the significant correlations between immune-related genes and microorganisms in both subtypes. The results showed that there were 25 immune-related genes in the C1 subtype that were related to at least one microorganism and 26 immune-related genes in the C2 subtype (Fig. S1A and B). Afterwards, 182 CTL genes were analyzed for associations with microorganisms. In the C1 subtype, 20 CTL-related genes were associated with at least one microorganism; in the C2 subtype, 19 CTL-related genes were associated with at least one microorganism (Fig. S2A and B).

To further explore the relationship between intratumoral microbiome and immunotherapy efficacy, we performed Pearson correlation analysis on the infiltration levels and abundances in the immunotherapy response and non-response groups. The results showed that in the TIDE response group, the infiltration level of Tregs was negatively correlated with microbial abundance, while the infiltration of eosinophils and neutrophils was positively correlated with microbial abundance. In the non-responsive group, there was a positive correlation between the three immune cell types and microbial abundance (Fig. 7B). Besides, the infiltration levels of macrophages M2 exhibited a significant difference between the responder and non-responder groups (Fig. 7C).

### Differential expression analysis between subtypes

**Fig 8:**
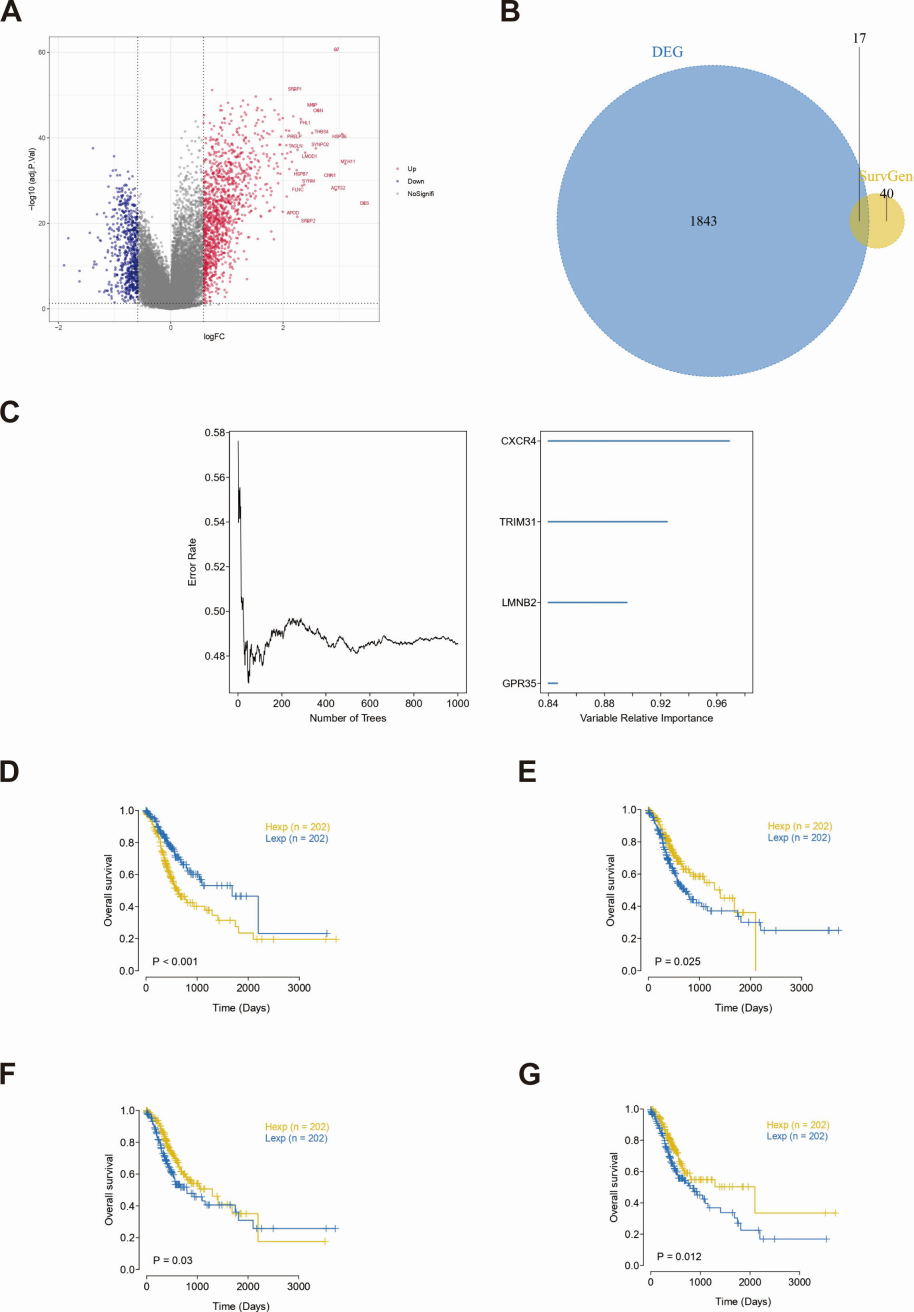
Acquisition of key genes and their survival analysis. (**A**) Volcano plot of differentially expressed genes was performed, with red indicating upregulated differential expression genes and blue indicating downregulated differential expression genes. (**B**) Venn diagram of the intersection between differential expression and prognostic genes was conducted. (**C**) Ranking of feature genes in random survival forests was carried out. Survival status of four key genes, namely, CXCR4 (**D**), GPR35 (**E**), LMNB2 (**F**), and TRIM31 (**G**).

Differences in expression levels were analyzed between the two subgroups (|logFC| > 0.585 and adj. *P*-value < 0.05) through the R package limma. Analysis of the differences between the C1 and C2 groups revealed 635 downregulated genes and 1,225 upregulated genes, for a total of 1,860 DEGs (Fig. 8A). Among them, 17 genes were prognostic genes for subtype analysis (Fig. 8B). To further identify the key genes affecting GC, we performed a random survival forest analysis on these 17 DEGs in the TCGA–STAD cohort, and we identified genes with relative importance > 0.8 as the final markers (Fig. 8C). The Kaplan–Meier curves of the four genes CXCR4, TRIM31, GPR35, and LMNB2 were significantly different (Fig. 8D through G).

### Regulatory network analysis of key genes

**Fig 9:**
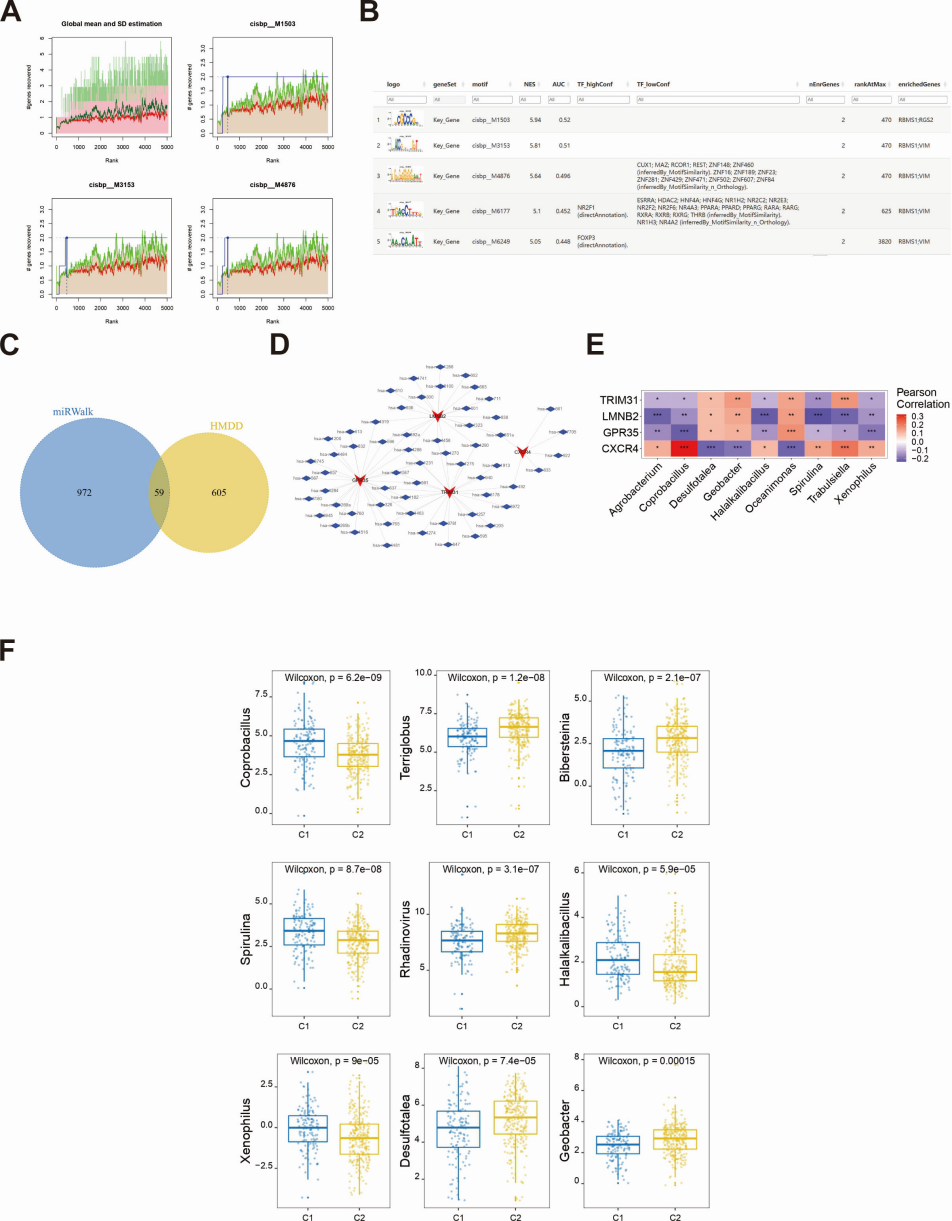
Regulatory network analysis of key genes. (**A**) Four motifs with high AUC, where the red line is the average recovery curve; the green line is the average plus standard deviation; and the blue line is the current motif’s recovery curve. The point where the current motif is farthest from the green line determines the maximum enrichment rank. (**B**) Top 5 enriched motifs and their corresponding transcription factors for the key genes were explored. (**C**) Fifty-nine disease-associated miRNAs were acquired. (**D**) The mRNA–miRNA interaction network was constructed. (**E**) Nine microbiomes with the highest significant correlation to key genes are shown. (**F**) The abundance values of microbiomes between subtypes were compared.

We used four key genes as the gene set for this analysis and found that they were regulated by common mechanisms, such as multiple transcription factors. Therefore, enrichment analysis of these transcription factors was performed using cumulative recovery curves (Fig. 9A). The results showed that the motif with the highest normalized enrichment score (NES: 5.94) was cisbp_M1503 (Fig. 9B). A total of two genes were enriched in this motif, namely, RBMS1 and RGS2. Table S2 shows all the enriched motifs and corresponding transcription factors of the key genes.

We also predicted potential miRNAs for four key genes using the miRWalk database. Initially, we extracted the mRNA–miRNA interaction pairs associated with these four key genes from the miRWalk database, identifying a total of 1,031 miRNAs, of which 59 were disease-associated miRNAs (Fig. 9C). Finally, we constructed an mRNA–miRNA interaction network using Cytoscape (v3.7) (Fig. 9D), which reveals the potential mRNA–miRNA interactions.

To further explore the mechanism of gene perturbation on disease progression, we performed a correlation analysis between the expression levels of key genes and intratumor microorganisms. We selected nine microorganisms with the most significant correlations with key genes for display. For example, *Agrobacterium*, *Coprobacillus*, and *Halalkalibacillus* showed a significant negative correlation with the genes GPR35, LMNB2, and TRIM31 and a significant positive correlation with the gene CXCR4 (Fig. 9E). Specific correlations between intratumoral microbiomes and key genes are shown in Fig. S3 to S6. Moreover, the abundances of microorganisms, such as *Terriglobus*, *Bibersteinia*, and *Rhadinovirus* in the C2 subgroup, were significantly greater than those in the C1 subgroup (Fig. 9F).

### Examination and validation of the immunological significance of key genes

**Fig 10:**
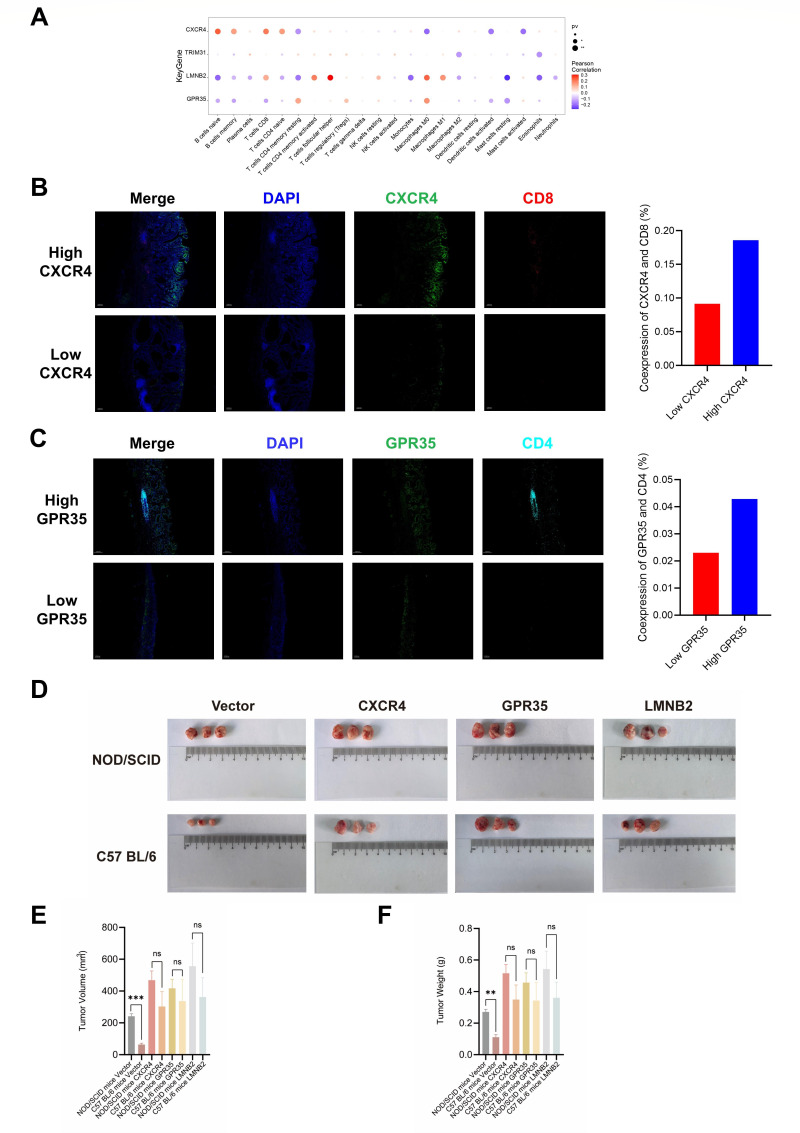
Exploration and validation of the immunological relevance of key genes. (**A**) Cibersort was performed to investigate the association between key genes and immune cells. Multiplex immunohistochemistry was used to validate the relationship between CXCR4 (**B**), GPR35 (**C**), and immune cells. (**D**) The mice subcutaneous tumor model was made to validate the influence of key genes on the function of the immune system. The tumor volume (**E**) and weight (**F**) were measured. Data are presented as mean ± standard deviation. ****P < 0.0001; ***P < 0.001; **P < 0.01; *P < 0.05; and ns *P* > 0.05.

We conducted a Pearson correlation analysis on the expression levels of key genes in tumor samples and the infiltration levels of immune cells. The CIBERSORT analysis revealed significant associations between CXCR4, GPR35, and LMNB2 with various immune cells, suggesting an important influence of key genes on the immune system (Fig. 10A). Given that T cells are the primary effector cells of the immune system, we validated the correlation between key genes and immune cells using mIHC. The results showed that CXCR4 (Fig. 10B) and GPR35 (Fig. 10C) were positively correlated with T cell markers CD8 and CD4 separately. In a subcutaneous tumor model in mice, tumors overexpressing key genes, such as CXCR4, GPR35, and LMNB2, exhibited a significantly accelerated proliferation compared to the control group (Fig. 10D). Considering the immune system’s role in tumor surveillance, the control cells showed a higher proliferation in the NOD/SCID mice compared to the C57 BL/6 mice. However, after overexpression of key genes, no significant differences were observed in the tumor volume (Fig. 10E) and weight (Fig. 10F) between the NOD/SCID and C57 BL/6 mice. These results suggest the relevance of key genes with immune cells and their potential role in immune evasion.

### Prognosis-related construction of prediction model and exploration of key intratumoral microbiota

**Fig 11:**
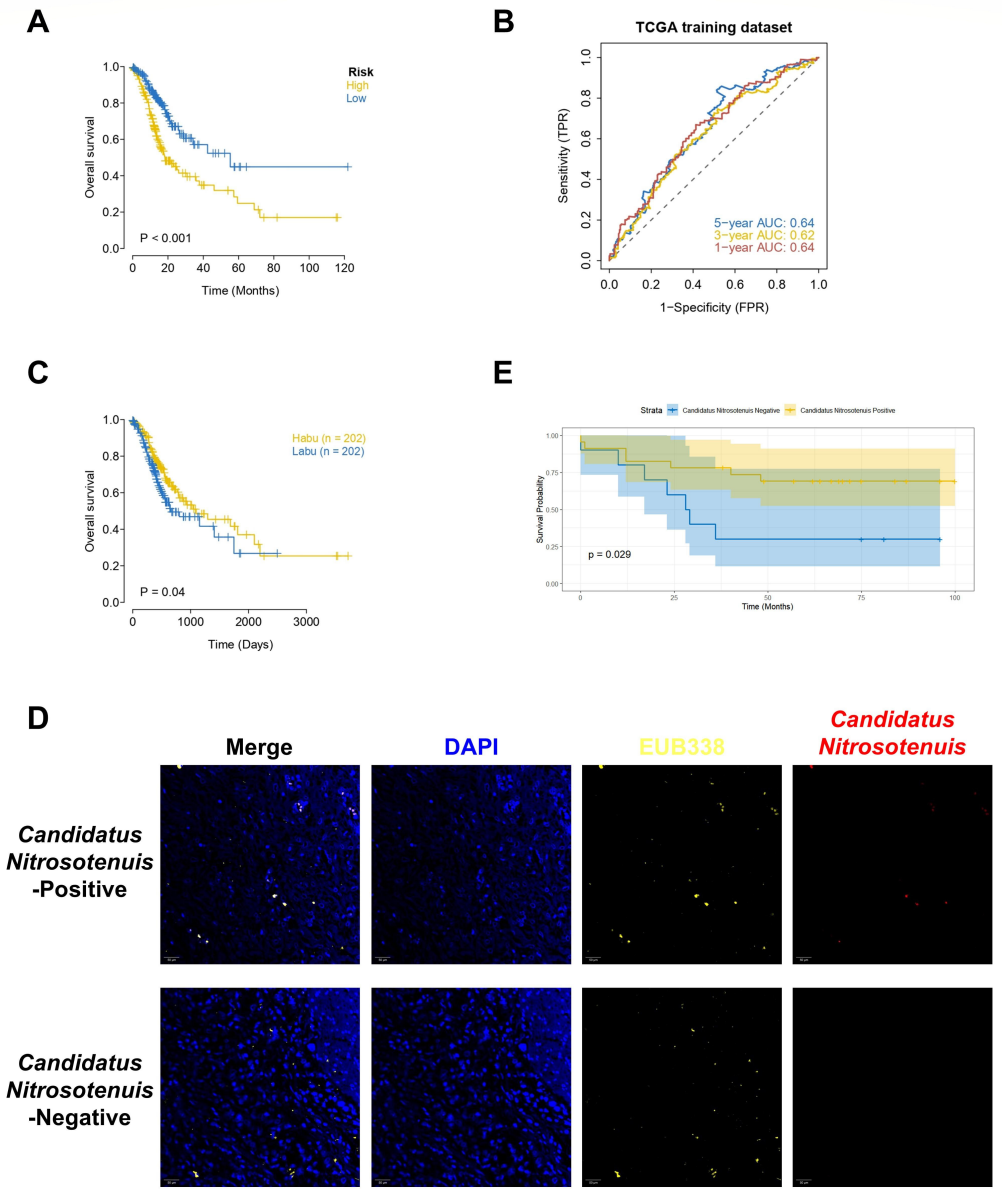
Prognosis-related construction of prediction model and exploration of key intratumoral microbiota construction of the prognostic model. The survival curve (**A**) and the ROC curve (1–3–5 years) (**B**) of The Cancer Genome Atlas (TCGA) model were conducted. (**C**) Survival curve of *Candidatus Nitrosotenuis* in TCGA-STAD. Habu: high abundance; Labu: low abundance. (**D**) FISH assay was used to validate the *Candidatus Nitrosotenuis* infection status in GC patients. (**E**) Kaplan–Meier survival analysis based on D was made.

To further identify the core genes in the key gene set, we collected clinical information from patients with STAD. Through the LASSO regression analysis, the best risk score for each sample was obtained for subsequent correlation analysis. Patients were divided into high- and low-risk groups according to the median risk score, and the Kaplan‒Meier curve analysis was used. The OS of patients in the high-risk group in the STAD cohort was significantly lower than that of patients in the low-risk group (Fig. 11A). In addition, the results of the ROC curve analysis revealed that the AUCs at 1, 3, and 5 years in the STAD cohort were not less than 0.6 (Fig. 11B).

Furthermore, we performed a Kaplan–Meier survival analysis on 53 intratumoral microbiotas associated with immune infiltration. The analysis indicated that *Candidatus Nitrosotenuis* exhibited a significant prognostic value in the TCGA–STAD cohort (Fig. 11C) (*P* = 0.04). Furthermore, we assessed the infection status of *Candidatus Nitrosotenuis* in the GC prognosis tissue microarray using FISH analysis (Fig. 11D) and conducted a Kaplan–Meier survival analysis. The results also demonstrated the significant role of *Candidatus Nitrosotenuis* in predicting the prognosis of GC patients (Fig. 11E) (*P* = 0.029), underscoring its critical role as an immune infiltration-related intratumoral microbiota in predicting GC prognosis.

## DISCUSSION

GC affects over 1 million people annually, ranking fifth in prevalence and fourth in cancer mortality (16). Consequently, the early diagnosis and management of GC are imperative. Immunophenotyping focusing on TME immune cell infiltration is vital for GC stratification and immunotherapy integration (17, 18). This study identified two GC immune subtypes using the TCGA and NCBI GEO data, C1 and C2, with C2 showing significantly better survival. Validation in another cohort confirmed these subtype differences, highlighting the importance of immunomolecular subtyping in GC research and treatment.

The immune cell type and abundance of TME impact tumor progression and immunotherapy response (19–21). However, the specific characteristics of immune cell infiltration within GC TME and their correlation with GC prognosis and immunotherapy response rates remain elusive. Our study found that GC subtype C1 had higher immune and stromal scores, with increased PDCD1LG2 and TLR9 expressions, suggesting greater immunotherapy sensitivity. In contrast, subtype C2 had more TNN gene mutations, higher neo-antigen levels, and mutation burden, enhancing immunogenic potential and immune system surveillance. This aligns with part of Ren’s view regarding the TME in GC (22) and also suggests C2’s better response to future therapies like vaccines or personalized immunotherapy. Furthermore, the elevated mutational burden in future therapeutic strategies, such as novel vaccines or personalized immunotherapy, could yield superior treatment outcomes (23, 24). C2 also showed better chemotherapy sensitivity, potentially leading to a more favorable prognosis due to the TME and immune system’s comprehensive effects on tumor growth.

Our study included GO and KEGG pathway analyses for GC subtypes C1 and C2, showing that C2 had higher scores in pathways related to protein synthesis, energy metabolism, cell cycle regulation, and DNA repair. Previous studies have shown that accurate segregation of sister chromatids plays a pivotal role in the faithful replication of genetic material and the prevention of genetic mutations (25, 26). Similarly, the TCA cycle contributes to the cellular metabolic energy supply (27, 28), while homologous recombination facilitates the repair of DNA double-strand breaks, thereby preserving genomic integrity and stability (29, 30). The elevated pathway scores in C2 suggest enhanced metabolic activity, effective DNA repair, precise cell cycle control, and superior protein synthesis, offering metabolic stability and prevention of cancer progression. These factors are likely to contribute to a better prognosis in C2 subtype patients.

Previous research mainly focused on gut microbiome interactions with cancer, but intratumoral microbiome–host interactions are less understood (31–33). Recent studies show intratumoral microbiota’s critical role in solid tumor pathogenesis, promoting tumor development through increased mutation rates, oncogenic pathway modulation, inflammation, and immune microenvironment changes (33–40). Research on the role of the intratumoral microbiota in GC and its contribution to GC progression remains limited. In GC, beyond *H. pylori*, diverse intratumoral microbes contribute to pathogenesis, varying by GC molecular subtypes (12, 41, 42). For instance, analyses by Abate et al. (11) and Yue et al. (14) reported the unique microbial diversity within GC and its potential role in promoting gastric adenocarcinoma progression through interactions with host epigenetic mechanisms. Our study analyzed microbial abundance in GC immune subtypes C1 and C2, finding associations with immune cell and gene expression differences. We hypothesize that intratumoral microbiota interacts with immune cells and genes, altering the local immune microenvironment and influencing GC progression.

To test the hypothesis that intratumoral bacteria affect the local immune microenvironment and GC progression, we analyzed the impact of microbial abundance on immune therapy responses in GC patients. We found significant correlations between microbial abundance and immune cell levels, including regulatory T cells, eosinophils, neutrophils, and macrophages M2, affecting therapy efficacy. *Candidatus Nitrosotenuis* plays a significant role in predicting the prognosis of GC patients. Shi et al. highlighted the effect of intratumoral accumulation of the gut microbiota on promoting immunotherapy efficacy (43). Key genes like CXCR4, TRIM31, GPR35, and LMNB2 were identified as prognostic markers, showing varying correlations with microbial abundance across immune subtypes. For example, a positive correlation was observed between specific bacteria (*Agrobacterium*, *Coprobacillus*, *Halalkalibacillus*) and CXCR4 expression, suggesting interactions between microbes and host genes (44). This finding suggests a complex interaction between microbial presence and host gene expression, potentially influencing or being influenced by tumor molecular characteristics. We also further validated the correlation between key genes and immune cells, as well as their impact on the host immune system, through bioinformatics analysis and experimental validations. Through these analyses, we deduced that intratumoral microbiomes engage in GC development by modulating immune infiltration and interacting with key host genes, a topic that merits further investigation. We also created a prognostic risk model based on these genes, which effectively predicted GC prognosis, with AUC values for 1-, 3-, and 5-year survival rates above 0.6, indicating its potential utility in GC prognosis prediction.

Our study offers a new perspective for GC molecular typing and personalized treatment. Future validation and clinical application of these molecular subtypes could enhance GC diagnosis and treatment. Additionally, deeper insights into intratumoral microorganisms’ role in GC molecular characteristics and treatment responses may lead to improved immunotherapy and microbial regulation strategies, enhancing patient outcomes and quality of life.

In this study, we identified two GC molecular subtypes through Cox univariate regression and NMF consensus clustering, noting differences in prognosis, immune characteristics, and drug sensitivity. We also explored immune cell types, immune pathway activation, key gene associations with disease progression, and microbial abundance, offering insights into GC pathogenesis.

### Limitations of the study

First, the findings of this study may have certain limitations due to the constraints of the analytical methods and the sources of the data sets; therefore, future research may require the adoption of broader approaches for more comprehensive investigations. Second, this study did not further validate the predictive models constructed based on immune factors, and we plan to confirm these results in future research. Finally, various external factors, such as diet, geographic microbiome variability, and host genetics, may influence TME. We will conduct more in-depth analyses in future studies to better explore the role of immune-related intratumoral microbiomes in the pathogenesis of GC.

## SUPPLEMENTAL MATERIAL

Supplemental Figures spectrum.02830-24-s0001.pdf Figures S1 to S6.

Table S1 spectrum.02830-24-s0002.xlsx Prognostic information of the gastric cancer prognosis tissue microarray.

Table S2 spectrum.02830-24-s0003.xlsx Enriched motifs and corresponding transcription factors of the key genes.

ASM does not own the copyrights to Supplemental Material that may be linked to, or accessed through, an article. The authors have granted ASM a non-exclusive, world-wide license to publish the Supplemental Material files. Please contact the corresponding author directly for reuse.
