# Supplementary material for: Prognostic implications of the interaction between intratumoral microbiome and immune response in gastric cancer
Source: Microbiol Spectr. 2025 Apr 9;13(5):e02830-24. doi: 10.1128/spectrum.02830-24 (PMC12054076; doi:10.1128/spectrum.02830-24)
Supplement: Supplemental Figures — Figures S1 to S6. [file spectrum.02830-24-s0001.pdf]

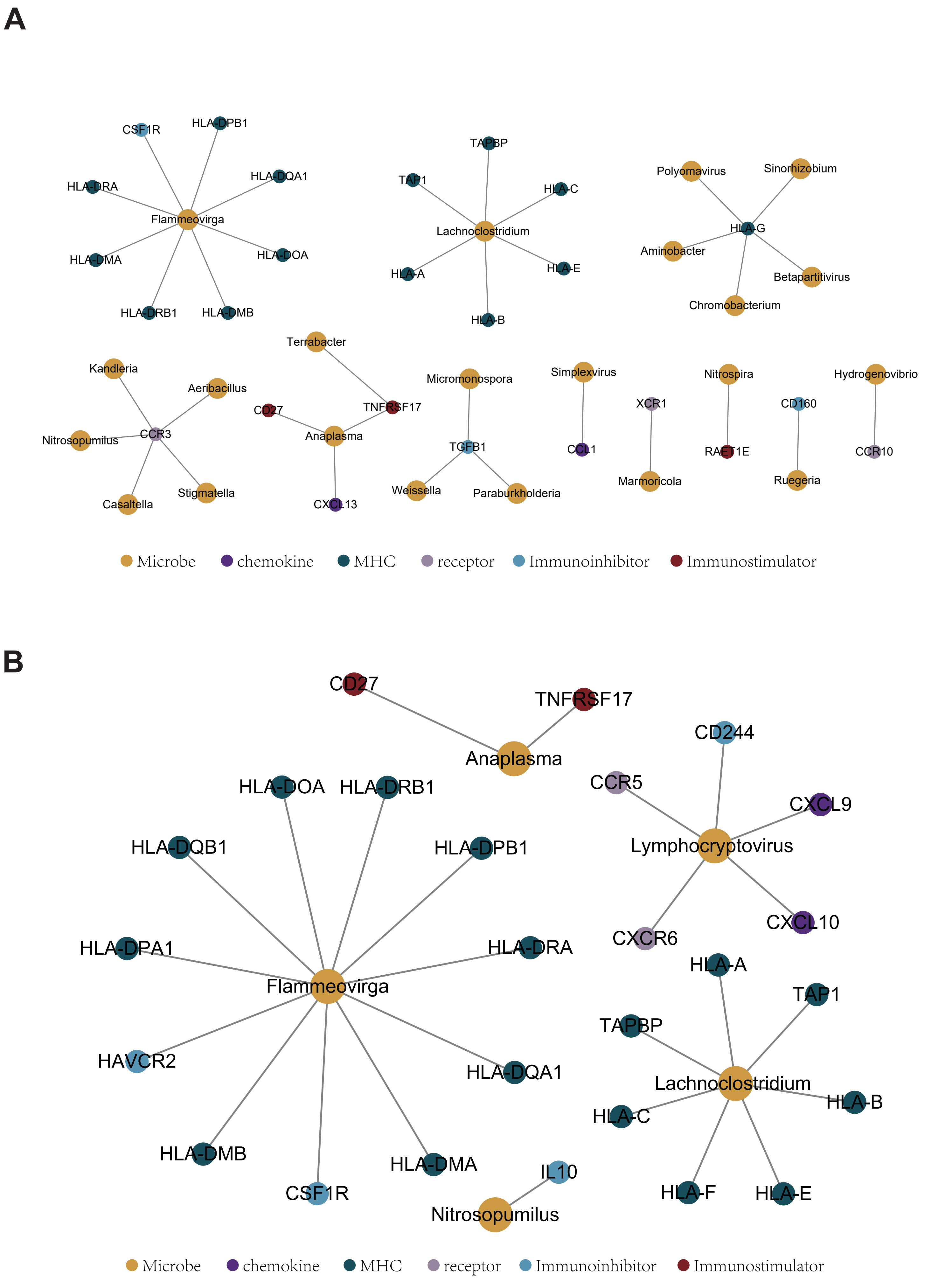

Fig. S1 Correlation analysis of immune genes with microbiomes in GC in C1 (**A**) and C2 (**B**) subtypes.

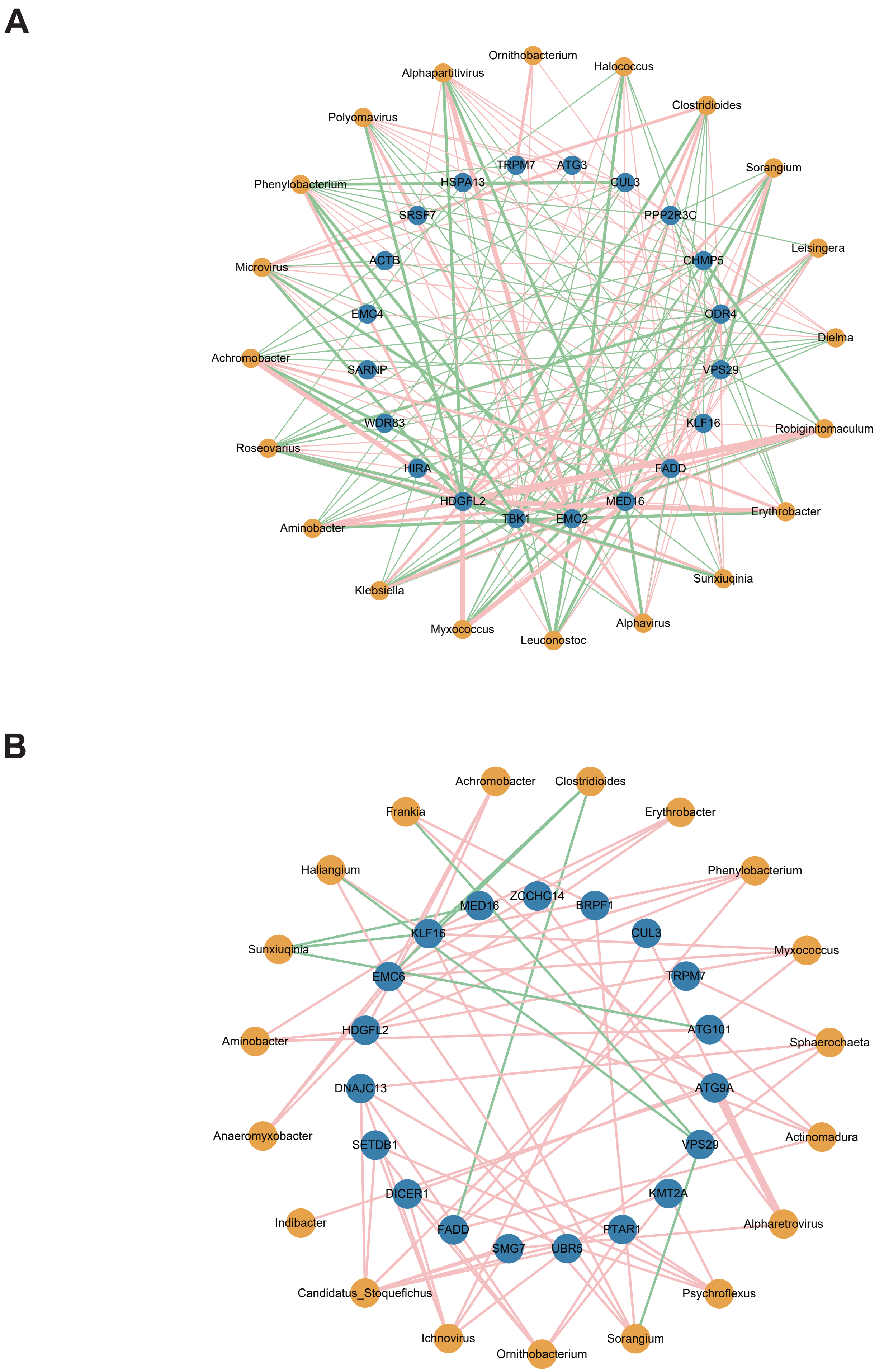

Fig. S2 Correlation analysis of cytotoxic T lymphocytes genes with microbiomes in GC in C1 (**A**) and C2 (**B**) subtypes. The thickness of the lines positively correlates with the strength of the relationship, with green representing negative correlations and pink representing positive correlations.

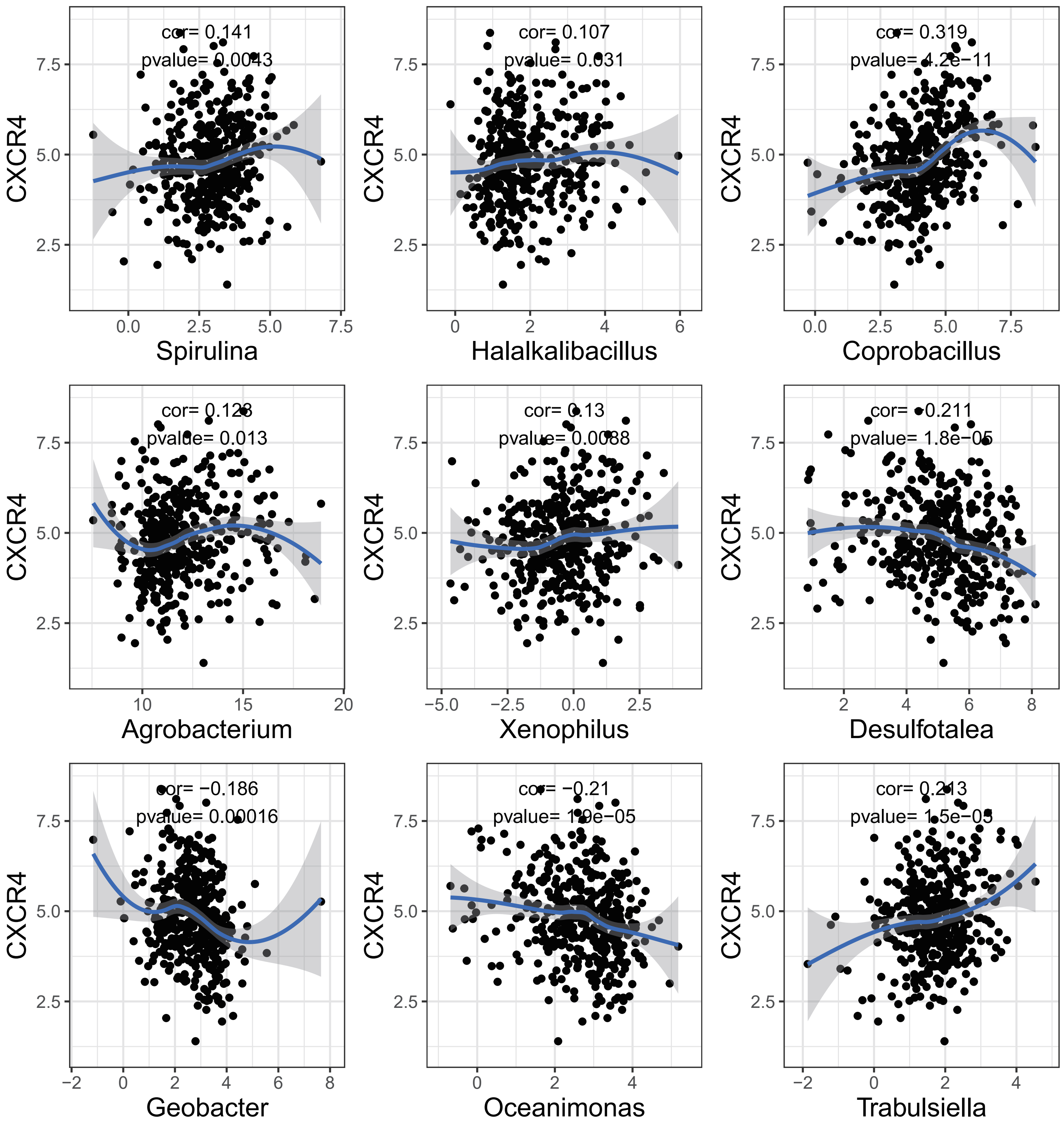

Fig. S3 Correlation analysis of CXCR4 with intratumoral microbiomes in GC.

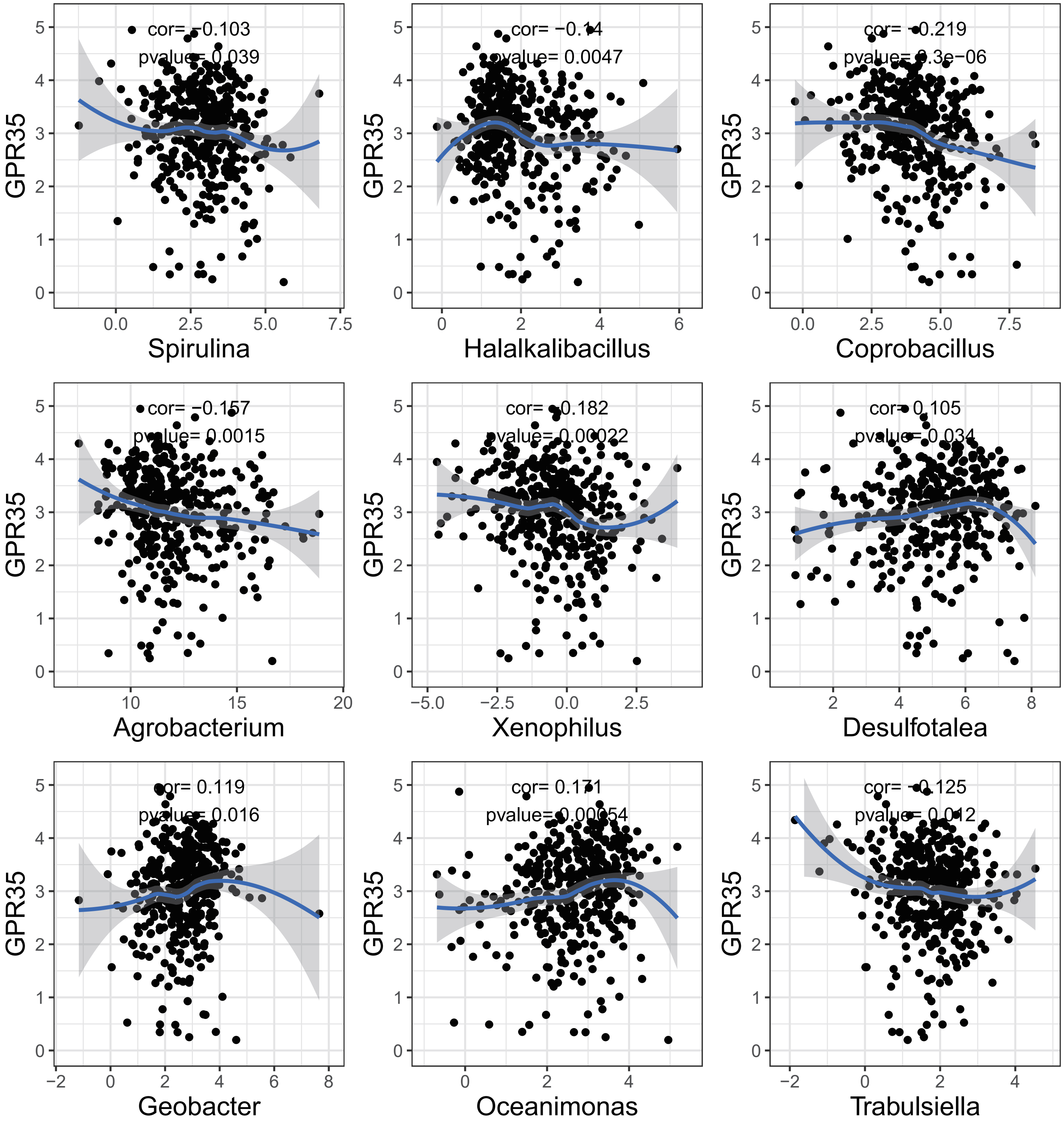

Fig. S4 Correlation analysis of GPR35 with intratumoral microbiomes in GC.

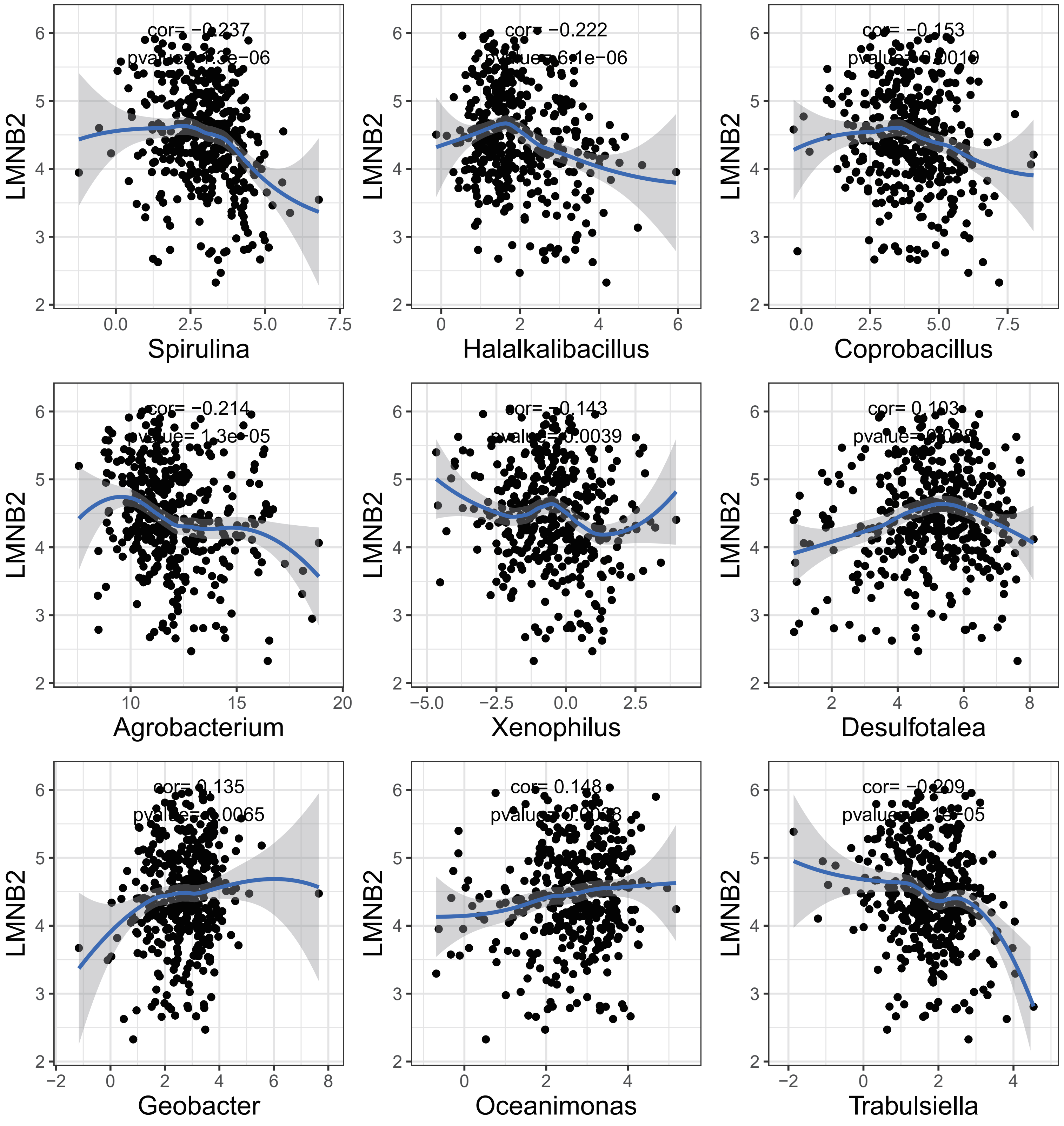

Fig. S5 Correlation analysis of LMNB2 with intratumoral microbiomes in GC.

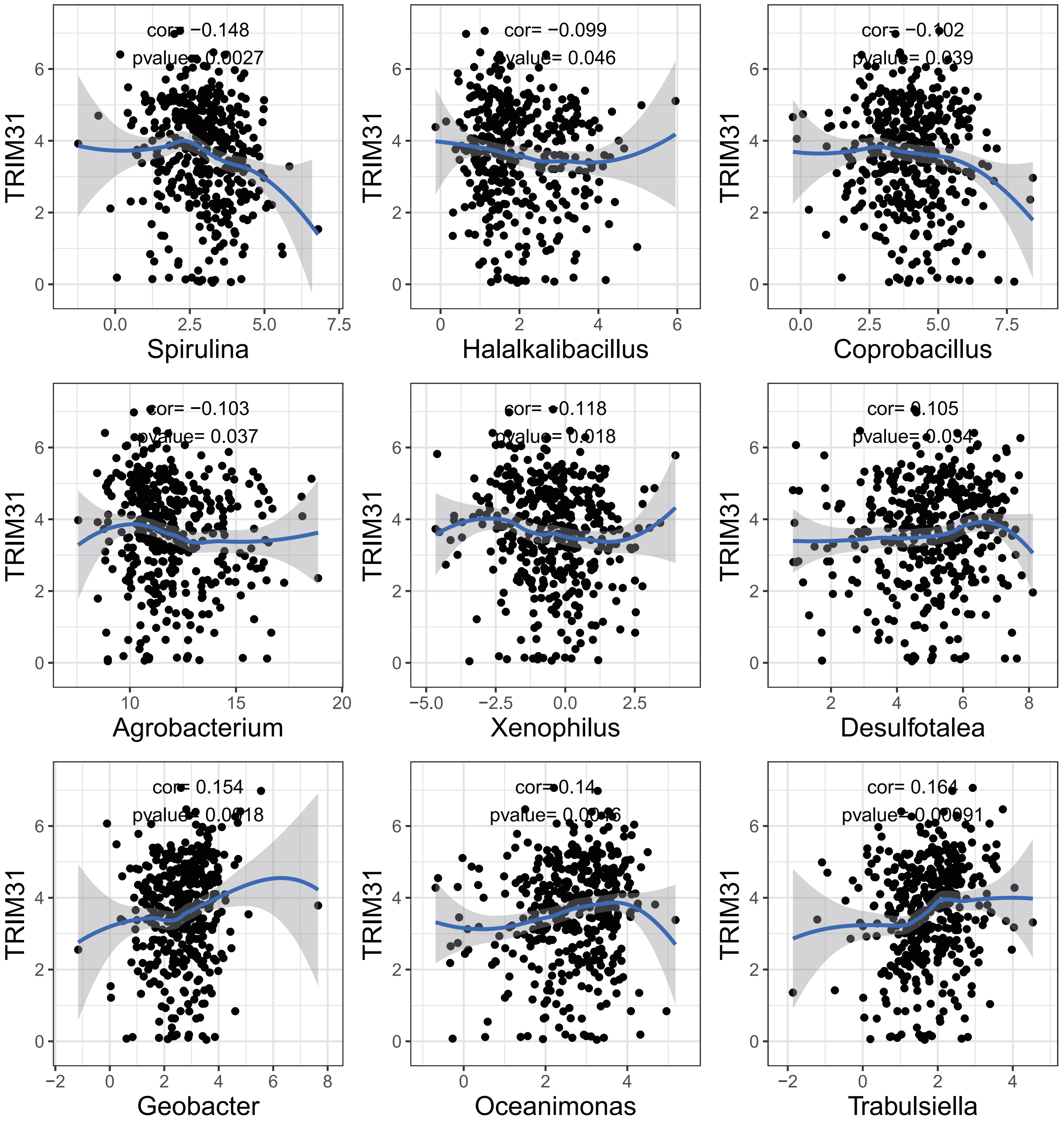

Fig. S6 Correlation analysis of TRIM31 with intratumoral microbiomes in GC.
